# Evidence for Two Modes of Synergistic Induction of Apoptosis by Mapatumumab and Oxaliplatin in Combination with Hyperthermia in Human Colon Cancer Cells

**DOI:** 10.1371/journal.pone.0073654

**Published:** 2013-08-27

**Authors:** Xinxin Song, Seog-Young Kim, Yong J. Lee

**Affiliations:** 1 Department of Surgery, School of Medicine, University of Pittsburgh, Pittsburgh, Pennsylvania, United States of America; 2 Department of Pharmacology & Chemical Biology, School of Medicine, University of Pittsburgh, Pittsburgh, Pennsylvania, United States of America; University of Torino, Italy

## Abstract

Colorectal cancer is the third leading cause of cancer-related mortality in the world-- the main cause of death from colorectal cancer is hepatic metastases, which can be treated with isolated hepatic perfusion (IHP). Searching for the most clinically relevant approaches for treating colorectal metastatic disease by isolated hepatic perfusion (IHP), we developed the application of oxaliplatin concomitantly with hyperthermia and humanized death receptor 4 (DR4) antibody mapatumumab (Mapa), and investigated the molecular mechanisms of this multimodality treatment in human colon cancer cell lines CX-1 and HCT116 as well as human colon cancer stem cells Tu-12, Tu-21 and Tu-22. We showed here, in this study, that the synergistic effect of the multimodality treatment-induced apoptosis was caspase dependent and activated death signaling via both the extrinsic apoptotic pathway and the intrinsic pathway. Death signaling was activated by c-Jun N-terminal kinase (JNK) signaling which led to Bcl-xL phosphorylation at serine 62, decreasing the anti-apoptotic activity of Bcl-xL, which contributed to the intrinsic pathway. The downregulation of cellular FLICE inhibitory protein long isoform (c-FLIP_L_) in the extrinsic pathway was accomplished through ubiquitination at lysine residue (K) 195 and protein synthesis inhibition. Overexpression of c-FLIP_L_ mutant (K195R) and Bcl-xL mutant (S62A) completely abrogated the synergistic effect. The successful outcome of this study supports the application of multimodality strategy to patients with colorectal hepatic metastases who fail to respond to standard chemoradiotherapy that predominantly targets the mitochondrial apoptotic pathway.

## Introduction

Colorectal cancer, which causes approximately 10% of cancer deaths in the United States, is the third leading cause of cancer-related mortality in the world; death usually results from uncontrolled metastatic disease. Unfortunately, only 10-15% of initial colorectal liver metastases are considered resectable [1,2]. The unresectable cases of liver metastatic disease can be treated with isolated hepatic perfusion (IHP), which involves a method of complete vascular isolation of the liver to allow for multimodality treatment of liver tumors [3–6].

Mapatumumab (Mapa) is a fully human IgG1 agonistic monoclonal antibody which exclusively targets and activates death receptor 4 (DR4) with high specificity and affinity [7–9]. Briefly, Mapa binds to the cell surface of DR4 and triggers the extrinsic apoptotic pathway, mainly through the activation of the pro-apoptotic initiator caspase-8. However, phase II trials showed little or no clinical activity of single-agent Mapa in patients with advanced refractory colorectal cancer or non-small cell lung cancer [10,11]. Several possible molecular mechanisms have been suggested including mutation/defects in death receptors, the death-inducing signaling complex, capsases, proapoptotic proteins or overexpression of anti-apoptotic molecules [12–14]. Thus, there is a continuing and significant need to develop applicable strategies to increase Mapa’s efficacy.

Hyperthermia, a treatment often used with isolated hepatic perfusion (IHP), maximizes tumor damage while preserving the surrounding normal tissue [5,6,15]. Oxaliplatin, a commonly used chemotherapeutic agent for colon cancer, is thought to trigger cell death mainly by inducing platinum-DNA adduct [3,16–18]. We previously developed a multimodality treatment using oxaliplatin pretreatment in combination with Mapa and hyperthermia to treat human colon cancer [19]. However, IHP delivering high doses of chemotherapy or biologic therapy regionally requires a standard operative technique, continuous intraoperative leak monitoring, and an external veno-veno bypass circuit [20]. Thus oxaliplatin pretreatment is not achievable in the procedure of the IHP in clinics, and all components of the multimodality procedure need to be performed simultaneously.

In this study, we investigated the therapeutic potential of the clinically relevant multimodality treatment schedule oxaliplatin and hyperthermia in combination with Mapa on human colon cancer cell lines and colon cancer stem cells. We report here that the multimodality treatment can sensitize human colon cancer cells to Mapa-induced apoptosis by multiple molecular mechanisms of action via both the intrinsic apoptotic pathway and the extrinsic pathway.

## Materials and Methods

### Cell cultures

Human colorectal carcinoma CX-1 cells, which were obtained from Dr. J.M. Jessup (National Institutes of Health) [21], were cultured in RPMI-1640 medium (Gibco BRL) containing 10% fetal bovine serum (HyClone). The human colorectal carcinoma HCT116 cell lines kindly provided by Dr. B. Vogelstein (Johns Hopkins University) were cultured in McCoy’s 5A medium (Gibco-BRL) containing 10% fetal bovine serum [22]. Human colon cancer stem cells, Tu-22, Tu-12 and Tu-21 [23], were established by Dr. E. Lagasse (University of Pittsburgh) and cultured in DMEM/F12 medium (Gibco BRL) containing 0.5% fetal bovine serum (HyClone) and 1% insulin, transferrin, and selenium (I.T.S, Fisher Scientific). All the cells were kept in a 37°C humidified incubator with 5% CO_2_.

### Reagents and antibodies

Oxaliplatin, MG132, cycloheximide (CHX) and protease inhibitor cocktail were obtained from Sigma Chemical Co. Mapatumumab (Mapa) was from Human Genome Sciences (Rockville, MD, USA). JNK inhibitor (SP6001125) and G418 were from Calbiochem. Anti-Flag, anti-caspase 8, anti-caspase 9, anti-caspase 3, anti-ubiquitin, anti-PARP, anti-phosphorylated JNK/JNK and anti-Bcl-xL antibody were from Cell Signaling. Anti-p-Bcl-xL (S62) antibody was from Chemicon/Millipore. Anti-FLIP antibody (NF6) was from Enzo Life Sciences. Anti-actin antibody was from Santa Cruz.

### Treatment

Cells were exposed to hyperthermia (42^°^C) in the presence/absence of Mapa and oxaliplatin for 1 h, and then incubated at 37^°^C for 3 h or 23 h. For hyperthermia, cells were sealed with parafilm and placed in a circulating water bath (Thomas Scientific), which was maintained within 0.02^°^C of the desired temperature.

### Transient transfection and stable transfection

For transient transfection, cells were transfected with Lipofectamine 2000 (Invitrogen), and were treated 48 h after transfection. For stable transfection, cells stably overexpressing HA-Bcl-xL wild-type (WT) or mutant types were prepared by transfecting CX-1 cells with HA-Bcl-xL-WT, HA-Bcl-xL-S62A (Ser62Ala), and HA-Bcl-xL-S62D (Ser62Asp) and maintained in 500 μg/ml G418. CX-1-Bcl-xL S62A cells were transfected with pLenti-Flag-FLIP_L_ and stable clones were selected with blasticidin (10 µg/ml). Pools of 3 clones were used in the experiment.

MTS [3-(4,5-dimethylthiazol-2-yl)-5-(3-carboxymethoxyphenyl)-2-(4-sulfophenyl)-2H-tetrazolium, MTS] assays

MTS studies were carried out using the Promega CellTiter 96 AQueous One Solution Cell Proliferation Assay (Promega). CX-1 cells were grown in tissue culture- coated 96-well plates and treated as described in Results. Cells were then treated with the MTS/phenazine methosulfate solution for 1 h at 37°C. Absorbance at 490 nm was determined using an enzyme-linked immunosorbent assay plate reader.

### Annexin V binding

Cells were heated in the absence or presence of Mapa and harvested by trypsinization, washed with serum-free medium, and suspended in binding buffer (Annexin V-FITC Staining Kit, PharMingen). This cell suspension was stained with mouse anti-human Annexin V antibody and PI and immediately analyzed by flow cytometry.

### Quantitative reverse transcription-polymerase chain reaction (RT-PCR) analysis

Total RNA was extracted and purified from cultured cells using the RNeasy Mini Kit (Qiagen, Valencia, CA), according to the manufacturer’s instructions. The RNA was quantified by determining absorbance at 260 nm. Two μg of total RNA from each sample was reverse transcribed into cDNA using the High-Capacity cDNA Reverse Transcription Kit (Life Technologies, Inc.) in a volume of 20 μl. Quantitative PCR (qPCR) was carried out using Applied Biosystems inventoried TaqMan assays (20X Primer Probe mix) corresponding to CASP8 and FADD-like apoptosis regulator (CFLAR; assay ID Hs00153439_m1), and glyceraldehyde-3-phosphate dehydrogenase (GAPDH; assay ID Hs02758991_g1). All reactions were carried out with 2 X TaqMan Universal PCR Master Mix (Applied Biosystems) on an Applied Biosystems StepOne Plus Real-Time PCR System according to standard protocols.

### Immunoprecipitation

Briefly, cells were lysed in CHAPS lysis buffer with protease inhibitor cocktail (Calbiochem). 0.5-1 mg of lysate was incubated with 1.5 μg of anti-Flag/ubiquitin antibody or rabbit IgG (Santa Cruz) at 4^°^C overnight, followed by the addition of protein A-agarose beads (Santa Cruz) and rotation at room temperature for 2 h followed by immunoblot analysis.

### Immunoblot analysis

Cells were lysed with Laemmli lysis buffer and boiled for 10 min. Protein content was measured with BCA Protein Assay Reagent (Pierce, Rockford, IL, USA), separated by SDS-PAGE and electrophoretically transferred to nitrocellulose membrane. The nitrocellulose membrane was blocked with 5% nonfat dry milk in PBS-Tween-20 (0.1% v/v) for 1 h and incubated with primary antibody at room temperature for 2 h. Horseradish peroxidase conjugated anti-rabbit or anti-mouse IgG was used as the secondary antibody. Immunoreactive protein was visualized by the chemiluminescence protocol (ECL, Amersham, Arlington Heights, IL, USA). The densities of bands were analyzed using Gel-pro application from Media Cybernetics. Some of the Western blots in the same panels were not produced from the same blots and were stripped and reprobed with anti-actin antibody to normalize for differences in protein loading to ensure equal protein loading.

### [^35^S] Methionine incorporation analysis

Cells were treated with medium containing 4 µCi of [^35^S]-L-methionine and exposed to hyperthermia (42^°^C) in the presence/absence of oxaliplatin (10 µg/ml) for 1 h, and then incubated at 37^°^C for 3 h. Cells were solubilized with 1 ml of 0.25 N NaOH and completely lysed by pipetting gently. [^35^S] methionine incorporation was analyzed by Wallac 1409 Liquid Scintillation Counter (PerkinElmer, MA, USA). [^35^S]-L-methionine incorporation levels were calculated by normalizing the [^35^S]-L-methionine counts per minute, corrected for nonspecific background, to the total protein levels.

### Site-directed mutagenesis

Lys 106 to Arg (K106R) and K195R mutations of the plasmid pCR3.V64-Met-Flag-FLIP_L_, which was a gift from Dr. Jurg Tschopp (University of Lausanne), were introduced into the c-FLIP_L_ gene using fully complementary mutagenic primers (QuickChange site-directed mutagenesis kit from Agilent Technologies). The following mutagenizing oligonucleotides were used: sense 5'-GAGATTGGTGAGGATTTGGATAGATCTG-ATGTGTCCTCATTAAT-3' and antisense 5'-ATTAATGAGGACACATCAGATCTAT-CCAAATCCTCACCAATCTC-3' for K106R mutant, sense 5'-CAAGCAGCAATCCA-AAAGAGTCTCAGGGATCCTTCAAAT-3' and antisense 5'-ATTTGAAGGATCCCTGAG-ACTCTTTTGGATTGCTGCTTG-3' for K195R mutant. Mutants were confirmed by sequence analysis.

### Statistical analysis

Statistical analysis was carried out using Graphpad InStat 3 software (GraphPad Software). Data showing comparisons between two groups were assessed using the Student’s t test. Comparisons among more than two groups were done using ANOVA with the appropriate post hoc testing. Statistical significance is marked with asterisks (*, p<0.05 and **, p<0.01).

## Results

The multimodality treatment of oxaliplatin/Mapa/hyperthermia activates both intrinsic and extrinsic pathways in human colon cancer cells

**Figure 1:**
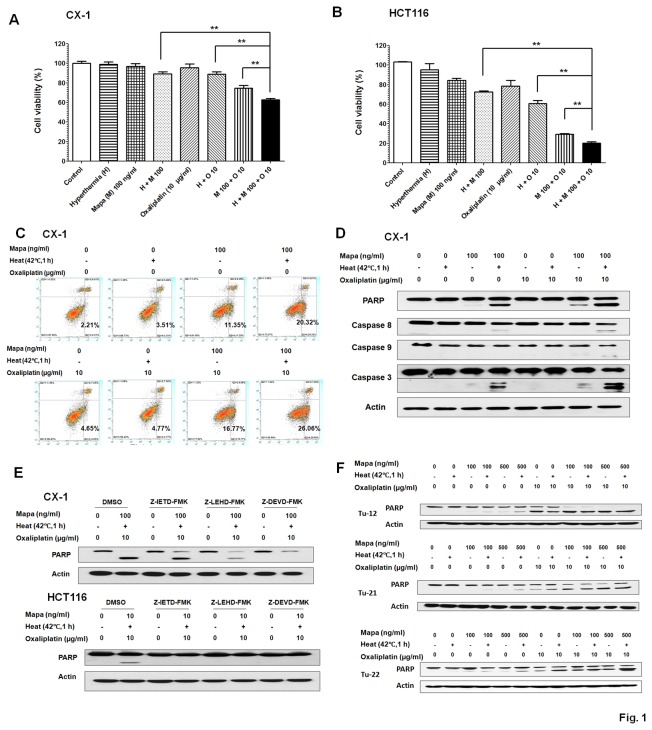
Effect of oxaliplatin and hyperthermia on Mapa-induced cytotoxicity and apoptosis. (A, B) CX-1 and HCT116 cells were exposed to normothermic or hyperthermic (42°C) conditions for 1 h in the presence/absence of Mapa and oxaliplatin and then incubated for 23 h at 37°C in the presence/absence of Mapa and oxaliplatin. Cell viability was analyzed by MTS assay. Error bars represent SD from triplicate experiments. Asterisk ** represents a statistically significant difference (P <0.01). (C) CX-1 cells were exposed to hyperthermia (42°C) for 1 h in the presence/absence of Mapa and oxaliplatin and then incubated for 3 h at 37°C in the presence/absence of Mapa and oxaliplatin. After treatment, cells were stained with fluorescein isothiocyanate (FITC)-Annexin V and propidium iodide (PI). Apoptosis was detected by the flow cytometric assay. (D) After treatment, the cleavage of caspase 8, caspase 9, caspase 3, or PARP was detected by immunoblotting. Actin was used to confirm the equal amount of proteins loaded in each lane. (E) CX-1 and HCT116 cells were treated with or without 20 µM Z-IETD-FMK (caspase 8 inhibitor), Z-LEHD-FMK (caspase 9 inhibitor), and Z-DEVD-FMK (caspase 3 inhibitor) for µmin followed by oxaliplatin/Mapa/hyperthermia and the cleavage of PARP was detected by immunoblotting. (F) Human colon cancer stem cells, Tu-12, Tu-21 and Tu-22, were exposed to normothermic or hyperthermic (42°C) conditions for 1 h in the presence/absence of Mapa and oxaliplatin at the indicated concentration and then incubated for 23 h at 37°C in the presence/absence of Mapa and oxaliplatin. PARP was detected by immunoblotting. Actin was used as loading control.

In this study, we attempted to develop clinically relevant multimodality therapy for colorectal metastatic disease which can be treated by IHP. The cell lines used include: human colorectal metastatic carcinoma HCT116 and CX-1 cells, and human colon cancer stem cells, Tu-12, Tu-21 and Tu-22, which were established by Dr. E. Lagasse (University of Pittsburgh) from the liver of metastatic colon cancer patients and cultured within the passages 10-30 [23]. Cancer stem cells (CSC) are able to self-renew, are tumorigenic, and are capable of producing the heterogeneous lineages of cancer cells that comprise the tumor. CSC should not only be affiliated with tumor initiation and growth, but are likely to be responsible for metastasis as well [24,25]. To investigate the effect of the multimodality treatment of oxaliplatin/Mapa/hyperthermia-induced cytotoxicity, cell viability was determined by MTS assay. As shown in Figure 1A and 1B, synergistic effect was observed in oxaliplatin/Mapa/hyperthermia compared with any other single treatment or bi-treatment in both cell lines (P <0.01). Figure 1C clearly shows that synergistic induction of apoptotic death occurred during treatment with oxaliplatin/Mapa/hyperthermia. Similar results were obtained in human colon cancer stem cells Tu-12, Tu-21 and Tu-22 (Figure 1F). These synergistic effects were due to an increase in the activation of caspases (Figure 1D). Figure 1D shows that 100 ng/ml Mapa resulted in a small amount of caspase 8 and 3 activation, and thus PARP cleavage (the hallmark feature of apoptosis). Interestingly, hyperthermia promoted the activation of caspase 8, while oxaliplatin promoted caspase 9 activation. Moreover, the synergistic effect of the multimodality treatment was blocked by Z-IETD-FMK (caspase 8 inhibitor), Z-LEHD-FMK (caspase 9 inhibitor), and Z-DEVD-FMK (caspase 3 inhibitor) in both cell lines (Figure 1E), indicating that both pathways played an important role in the synergistic effect of the multimodality treatment.

### Dose responses of oxaliplatin and hyperthermia on Mapa-induced apoptosis

**Figure 2:**
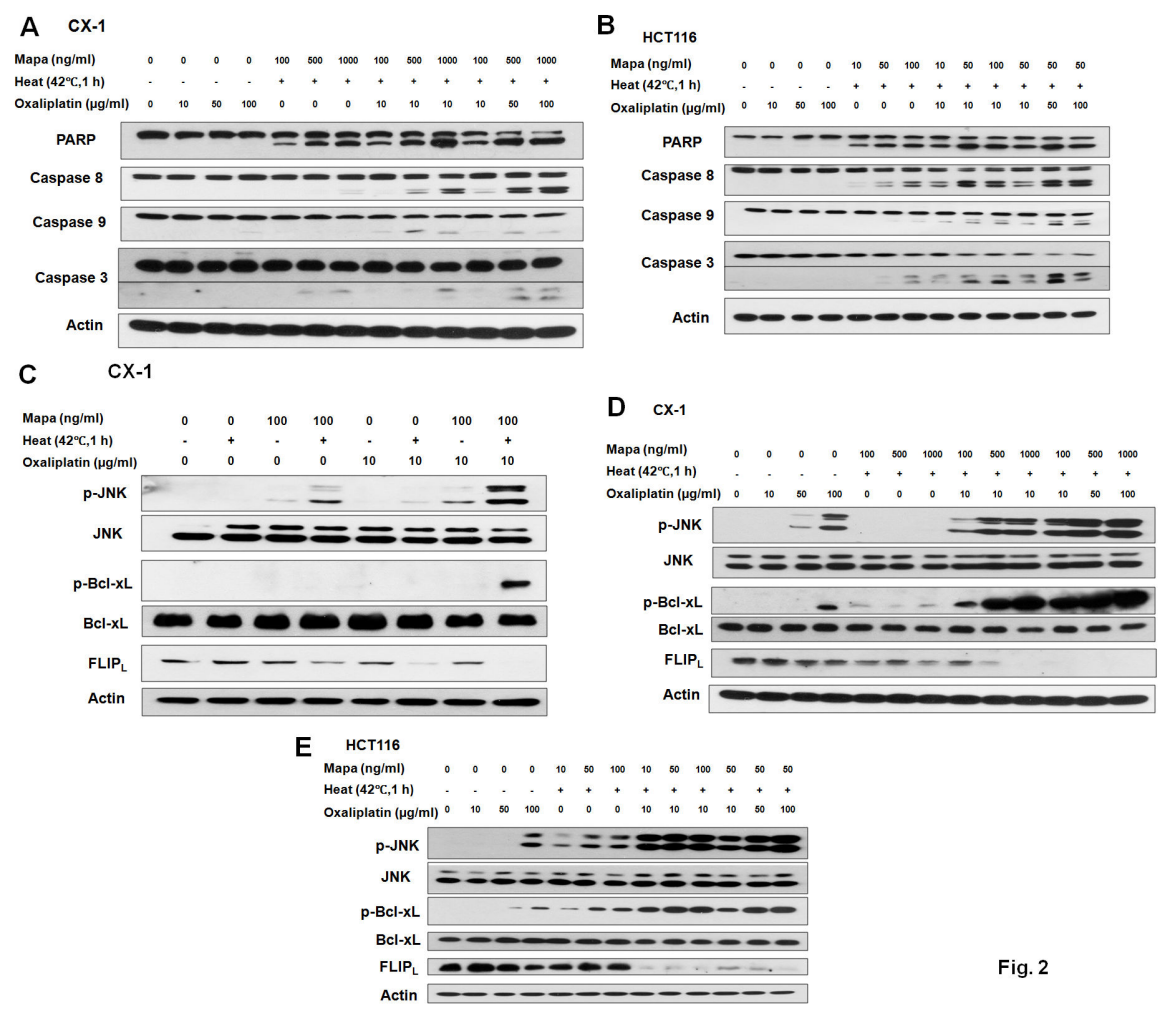
Multimodality treatment-induced JNK phosphorylation, Bcl-xL phosphorylation and reduction in c-FLIP_L_ level. (A) CX-1 and (B) HCT116 cells were exposed to hyperthermia (42°C) for 1 h in the presence/absence of Mapa and oxaliplatin and incubated for 3 h at 37°C in the presence/absence of Mapa and oxaliplatin. After treatment, the cleavage of caspase 8, caspase 9, caspase 3, or PARP was detected by immunoblotting. Actin was used to confirm the equal amount of proteins loaded in each lane. (C) CX-1 cells were exposed to hyperthermia (42°C) for 1 h in the presence/absence of 100 ng/ml Mapa and 10 µg/ml oxaliplatin and then incubated for 3 h at 37°C in the presence/absence of Mapa and oxaliplatin. After treatment, cells were immunoblotted with anti-phospho-JNK/JNK, anti-phospho-Bcl-xL/Bcl-xL and anti-FLIP antibodies. (D) CX-1 cells were exposed to hyperthermia (42°C) for 1 h in the presence/absence of Mapa (100 ng/ml-1000 ng/ml) and oxaliplatin (10 µg/ml-100 µg/ml) and incubated for 3 h at 37°C in the presence/absence of Mapa and oxaliplatin. After treatment, phospho-JNK/JNK, phospho-Bcl-xL/Bcl-xL and FLIP_L_ were detected by immunoblotting. Actin was used to confirm the equal amount of proteins loaded in each lane. (E) HCT116 cells were exposed to hyperthermia (42°C) for 1 h in the presence/absence of Mapa (10 ng/ml-100 ng/ml) and oxaliplatin (10 µg/ml-100 µg/ml) and then incubated for 3 h at 37°C in the presence/absence of Mapa and oxaliplatin. After treatment, phospho-JNK/JNK, phospho-Bcl-xL/Bcl-xL and FLIP_L_ were detected by immunoblotting. Actin was used to confirm the equal amount of proteins loaded in each lane.

We observed that as the doses of Mapa and oxaliplatin increased, caspase 8/9/3 activation and PARP cleavage were enhanced, indicating that the synergistic effect of the multimodality treatment-induced apoptosis was dose dependent (Figure 2A). Furthermore, our results suggest that both the intrinsic and extrinsic apoptotic pathways were involved in the synergistic effect of the multimodality treatment. Similar data was obtained in HCT116 cells (Figure 2B).

### Multimodality treatment-induced JNK activation, Bcl-xL phosphorylation and reduction in c-FLIP_L_ level

To further understand the mechanisms of how the intrinsic and extrinsic pathways were involved in the multimodality treatment-induced apoptosis, we examined Bcl-xL as well as c-FLIP. Figure 2C shows that there was no change in the amount of Bcl-xL protein, but the phosphorylation of Bcl-xL dramatically increased, accompanied by JNK phosphorylation. In addition, the level of c-FLIP_L_ significantly decreased during the multimodality treatment in CX-1 cells. Figure 2D demonstrates that JNK was activated and Bcl-xL was phosphorylated at serine 62 in a dose-dependent manner in CX-1 cells. Interestingly, the level of c-FLIP_L_ dramatically decreased when oxaliplatin was combined with hyperthermia. Similar data was obtained in HCT116 cells (Figure 2E).

### The kinetics of multimodality treatment in CX-1 and HCT116 cells

**Figure 3:**
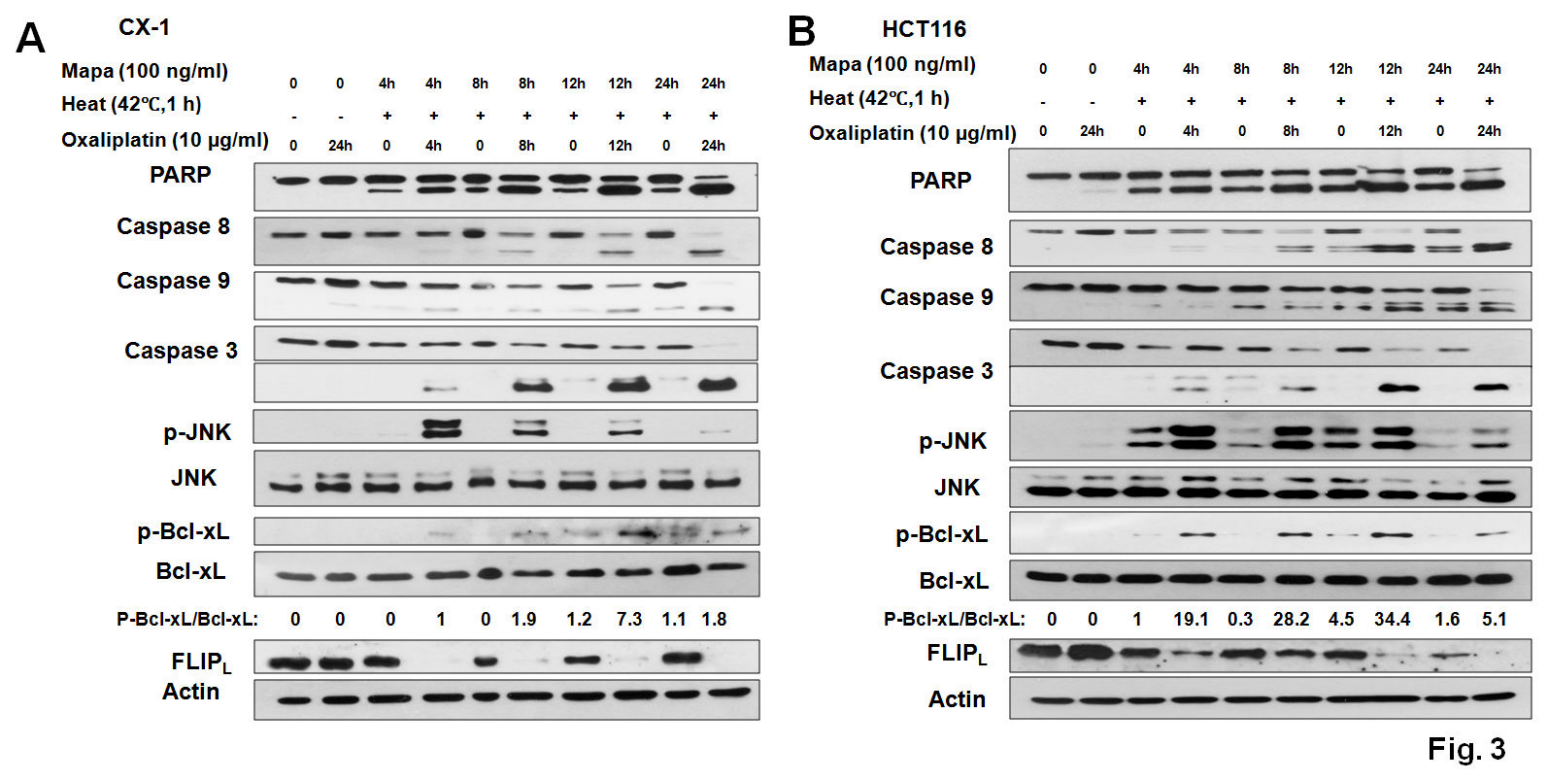
The kinetics of multimodality treatment in CX-1 and HCT116 cells. CX-1 (A) and HCT116 (B) cells were exposed to hyperthermic (42°C) conditions for 1 h in the presence/absence of Mapa and oxaliplatin and incubated at 37°C in the presence/absence of Mapa and oxaliplatin for 3 h, 7 h, 11 h and 23 h. After treatment, the cleavage of caspase 8/9/3 and PARP, phospho-JNK/JNK, phospho-Bcl-xL/Bcl-xL and FLIP_L_ were detected by immunoblotting. Actin was used to confirm the equal amount of proteins.

We observed that the effect of the multimodality treatment increased as time progressed in CX-1 (Figure 3A) and HCT116 cells (Figure 3B). JNK activation reached maximum at 4 h after the initial treatment and gradually decreased during the multimodality treatment. Data from immunoblot and imaging gel analyses show that Bcl-xL phosphorylation reached the peak around 12 h after the treatment indicating that JNK activation was an early event and might regulate the Bcl-xL phosphorylation. In CX-1 cells the level of c-FLIP_L_ dramatically decreased at 4 h after the treatment of oxaliplatin combined with hyperthermia, while in HT116 cells, it reached minimum 24 h after the treatment.

### The requirement of JNK activation and Bcl-xL phosphorylation in the multimodality treatment-induced apoptosis

**Figure 4:**
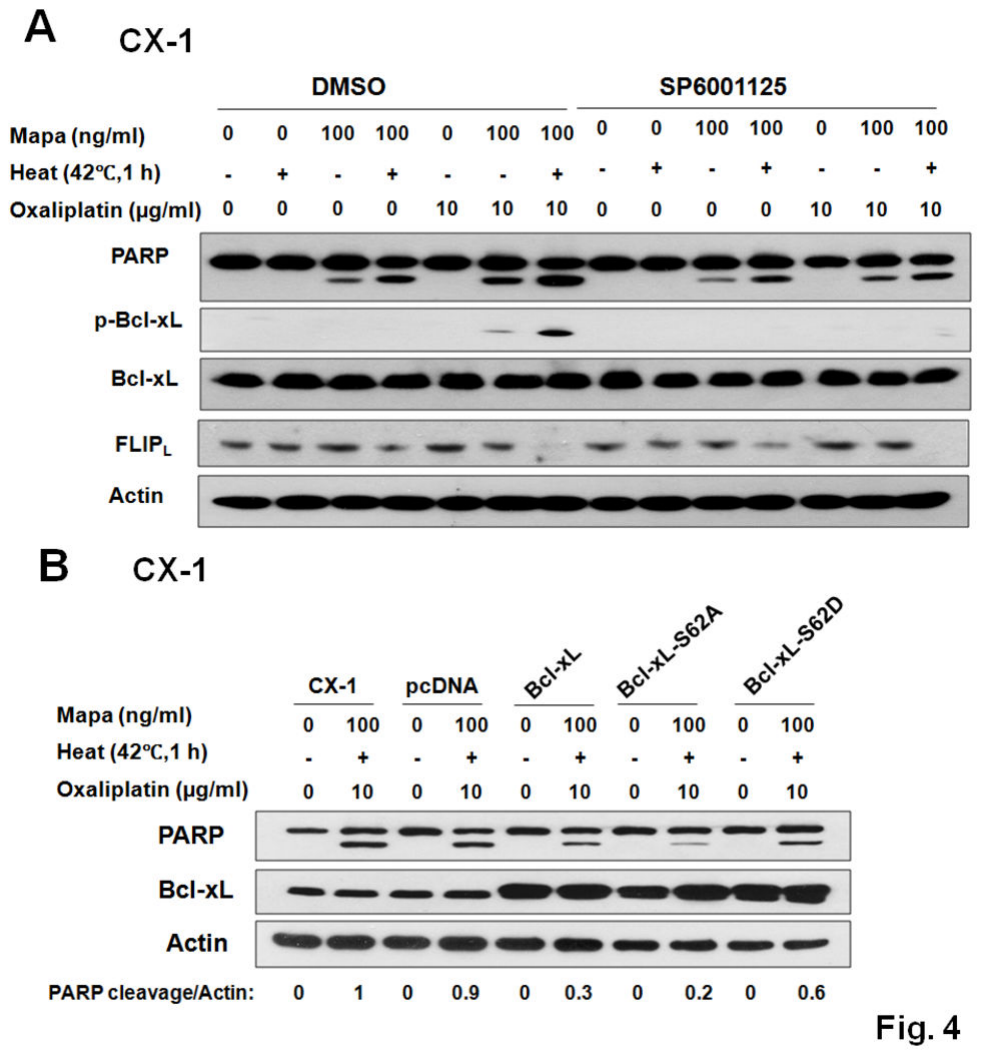
The requirement of phosphorylation of JNK and Bcl-xL in the multimodality treatment-induced apoptosis in CX-1 cells. (A) Cells were pretreated with JNK inhibitor 25 µM SP6001125 followed by oxaliplatin/Mapa/hyperthermia and immunoblotted with anti-PARP, anti-phospho-Bcl-xL and anti-Bcl-xL antibody. (B) Transfectants with control plasmid (pcDNA), wild-type Bcl-xL (Bcl-xL-WT), Ser62/Ala phospho-defective Bcl-xL mutant (Bcl-xL-S62A), or Ser62/Asp phospho-mimic Bcl-xL mutant (Bcl-xL-S62D) were treated with oxaliplatin/Mapa/hyperthermia and immunoblotted with anti-PARP or anti-Bcl-xL antibody. Actin was used to confirm the equal amount of proteins loaded in each lane.

JNK inhibitor SP6001125 partially reduced oxaliplatin/Mapa/hyperthermia-induced PARP cleavage in CX-1 cells, indicating that the JNK pathway was crucial for multimodality treatment-induced apoptosis (Figure 4A). Noticeably, SP6001125 highly reduced the level of Bcl-xL phosphorylation in CX-1 cells, which provides strong evidence that multimodality treatment-induced Bcl-xL phosphorylation requires JNK activation. Zhao et al. reported that JNK activation mediates c-FLIP downregulation [26]. This possibility was examined in Figure 4A. We observed that no restoration of c-FLIPL occurred during treatment with SP6001125. This observation was consistent with other researchers’ reports [27,28].

To evaluate the effect of Bcl-xL phosphorylation at Ser62 on its anti-apoptotic activity, we established CX-1-derived cell lines stably overexpressing wild-type Bcl-xL (Bcl-xL-WT), Ser62Ala phospho-defective Bcl-xL mutant (Bcl-xL-S62A), Ser62Asp phospho-mimic Bcl-xL mutant (Bcl-xL-S62D), or the corresponding empty vector (pcDNA). As expected, overexpression of Bcl-xL-WT prevented oxaliplatin/Mapa/hyperthermia-induced PARP cleavage. Interestingly, overexpression of Bcl-xL-S62D enhanced PARP cleavage, whereas that of Bcl-xL-S62A inhibited PARP cleavage (Figure 4B). These data suggest that the level of Bcl-xL and its phosphorylation at S62 play an important role in the multimodality-induced apoptosis.

### Reduction in c-FLIP_L_ level following hyperthermia and oxaliplatin in CX-1 cells

**Figure 5:**
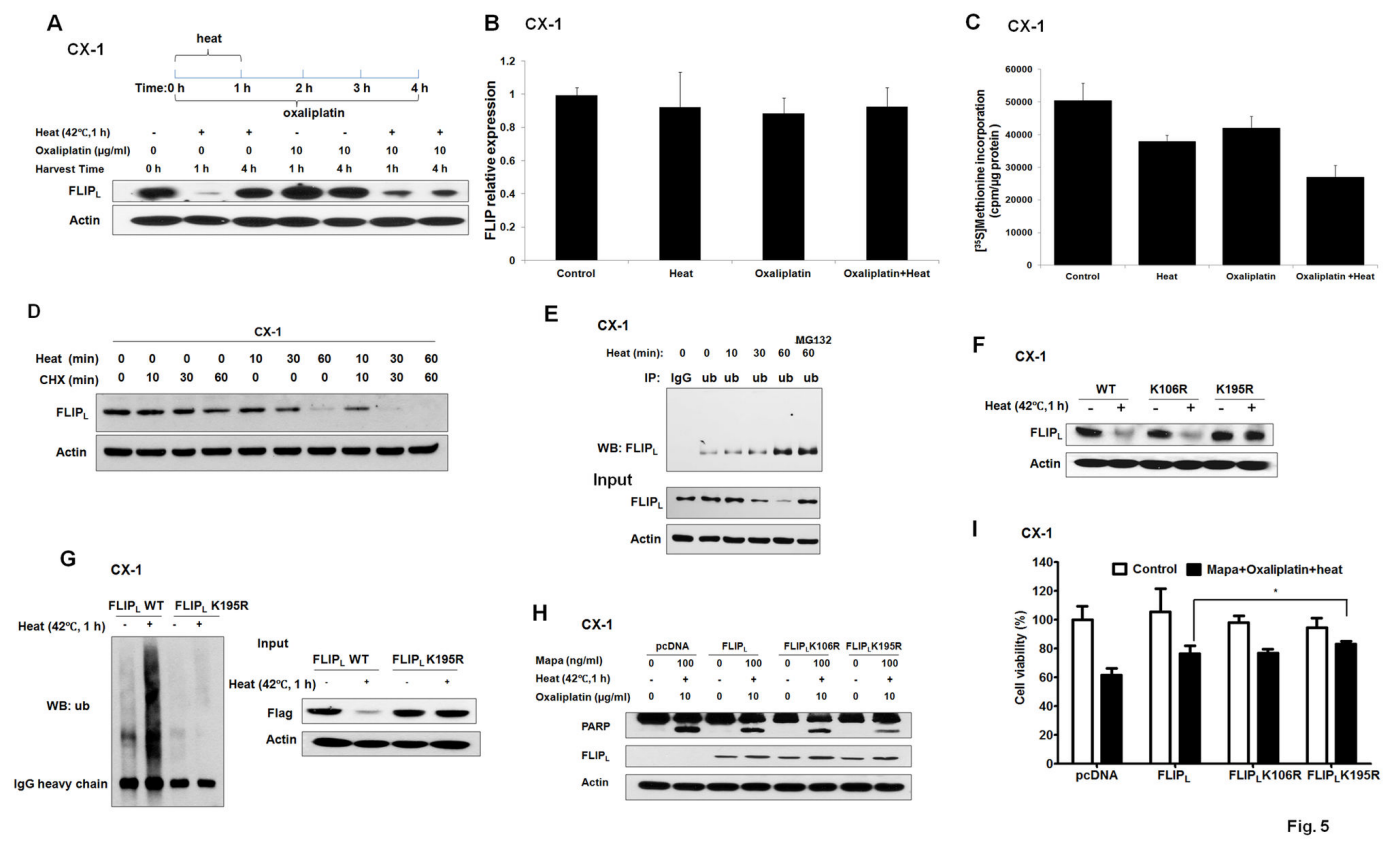
Reduction in c-FLIP_L_ level following hyperthermia and oxaliplatin in CX-1 cells. (A) Cells were exposed to 37°C or 42°C for 1 h in the presence/absence of 10 µg/ml oxaliplatin and then harvested immediately or 3 h after incubation at 37°C. c-FLIP_L_ was examined by Western blot analysis. (B) Cells were exposed to 37°C or 42°C for 1 h in the presence/absence of 10 µg/ml oxaliplatin and then incubated for 3 h at 37°C. Quantitative reverse transcription-polymerase chain reaction (qRT-PCR) was performed to measure relative c-FLIP mRNA level. The bar graph represents mean values (+SD) from triplicate experiments. (C) Cells were exposed to 37°C or 42°C for 1 h in the presence/absence of 10 µg/ml oxaliplatin and then incubated for 3 h at 37°C. Protein synthesis was measured by [^35^S] Methionine incorporation. (D) Cells were treated with 30 µg/ml CHX, or exposed to hyperthermia in the presence or absence of CHX. The levels of c-FLIP_L_ and loading control actin were measured by Western blot analysis. (E) Cells were exposed to hyperthermia for 30 or 60 min in the presence/absence of MG132. Lysate samples were immunoprecipitated with anti-ubiquitin antibody and protein G-Sepharose. The ubiquitinated FLIP was detected by Western blot with anti-FLIP antibody. (F) Cells were transiently transfected with 4 µg plasmid containing mock, K106R (106 lysine residue was replaced with arginine), K195R, or wild-type (WT) c-FLIP_L_. After 48 h incubation, cells were exposed to hyperthermia at 42°C for 1 h. The level of c-FLIP_L_ was detected by anti-FLIP antibody. Actin was used as an internal control. (G) Cells were transiently transfected with Flag-tagged c-FLIP_L_ WT or K195R plasmid; 48 h later, cells were subjected to hyperthermia at 42°C for 1 h. The levels of ubiquitinated c-FLIP_L_ were detected by IP with anti-Flag antibody followed by Western blot using anti-ubiquitin antibody. The presence of transfected c-FLIP_L_ in the lysates was verified by Western blot. Actin was shown as an internal standard. (H) Cells were transiently transfected with c-FLIP_L_ WT, K106R, or K195R plasmid; 48 h later, cells were heated at 42°C for 1 h in the presence/absence of Mapa (100 ng/ml) and oxaliplatin (10 µg/ml) and then incubated at 37^°^C for 3 h. Lysates containing equal amounts of protein were immunoblotted with anti-PARP and anti-FLIP antibody. Actin was shown as an internal standard. (I) Cells were transiently transfected with c-FLIP_L_ WT, K106R, or K195R plasmid; 48 h later, cells were heated at 42°C for 1 h in the absence or presence of Mapa (100 ng/ml) and oxaliplatin (10 µg/ml), and then incubated for 24 h at 37°C. Cell viability was analyzed by MTS assay. Error bars represent SD from triplicate experiments. Asterisk * represents a statistically significant difference (P <0.05).

c-FLIP is the major inhibitor of the extrinsic apoptotic pathway through inhibition of caspase-8 activation, and we observed that the level of c-FLIP_L_ was reduced after hyperthermia at 42°C for 1 h in CX-1 cells as shown in Figure 5A. However, c-FLIP_L_ was restored to the normal level after 3 h incubation at 37°C which was consistent with our previous paper [24]. Oxaliplatin (10 µg/ml, 4h) alone didn’t reduce the level of c-FLIP_L_. Interestingly, the level of c-FLIP_L_ was maintained at a reduced level when hyperthermia combined with oxaliplatin.

Quantitative RT-PCR showed that no significant inhibition of c-FLIP expression at the mRNA level was evident after hyperthermia, oxaliplatin, or the combination (Figure 5B). We observed in Figure 5C that 25% of protein synthesis was inhibited in hyperthermia, whereas there was 46% protein synthesis inhibition in oxaliplatin combined with hyperthermia. Figure 5D shows that reduction of c-FLIP_L_ level by 42°C for 1 h heating alone was more than that by 30 µg of cyclohexmide which inhibits protein synthesis by 99% [28]. These results suggest that protein synthesis inhibition alone is not a major factor for downregulation of FLIP_L_ by hyperthermia. Remarkably, c-FLIP_L_ was recovered during 3 h of normothermic condition, indicating that c-FLIP_L_ was resynthesized. However, the recovery was delayed by treatment with hyperthermia and oxaliplatin, as protein synthesis was significantly inhibited.

Figure 5E shows that the ubiquitination of endogenous c-FLIP_L_ increased upon hyperthermia treatments. Moreover, proteasome inhibitor MG132 blocked the degradation of c-FLIP_L_, confirming the existence of proteasomal-mediated degradation of the protein after hyperthermia.

The online software UbPred, which predicts protein ubiquitination sites, showed that lysine 106 and 195 had the highest scores. We replaced 106 and 195 lysine with arginine and tested the stability of the full-length c-FLIP_L_ carrying the resulting point mutation. As shown in Figure 5F, in the transfection group, c-FLIP_L_ K106R was easily degraded when subjected to hyperthermia while K195R was refractory to degradation by hyperthermia. Figure 5G confirms that c-FLIP_L_ wild-type (WT) was efficiently ubiquitinated but not the K195R mutant, which was found virtually without ubiquitination. We observed that c-FLIP_L_ K195R expressing cells were the most resistant to the multimodality treatment-induced apoptotic cell death (Figure 5H and 5I). These results suggest that the transient hyperthermia-mediated degradation of c-FLIP_L_ involved ubiquitination of K195, and K195R mutant conferred resistance against the multimodality treatment-induced apoptotic death.

### Abrogation of the synergistic effect by overexpression of c-FLIP_L_ K195R and Bcl-xL-S62A in CX-1 and HCT116 cells

**Figure 6:**
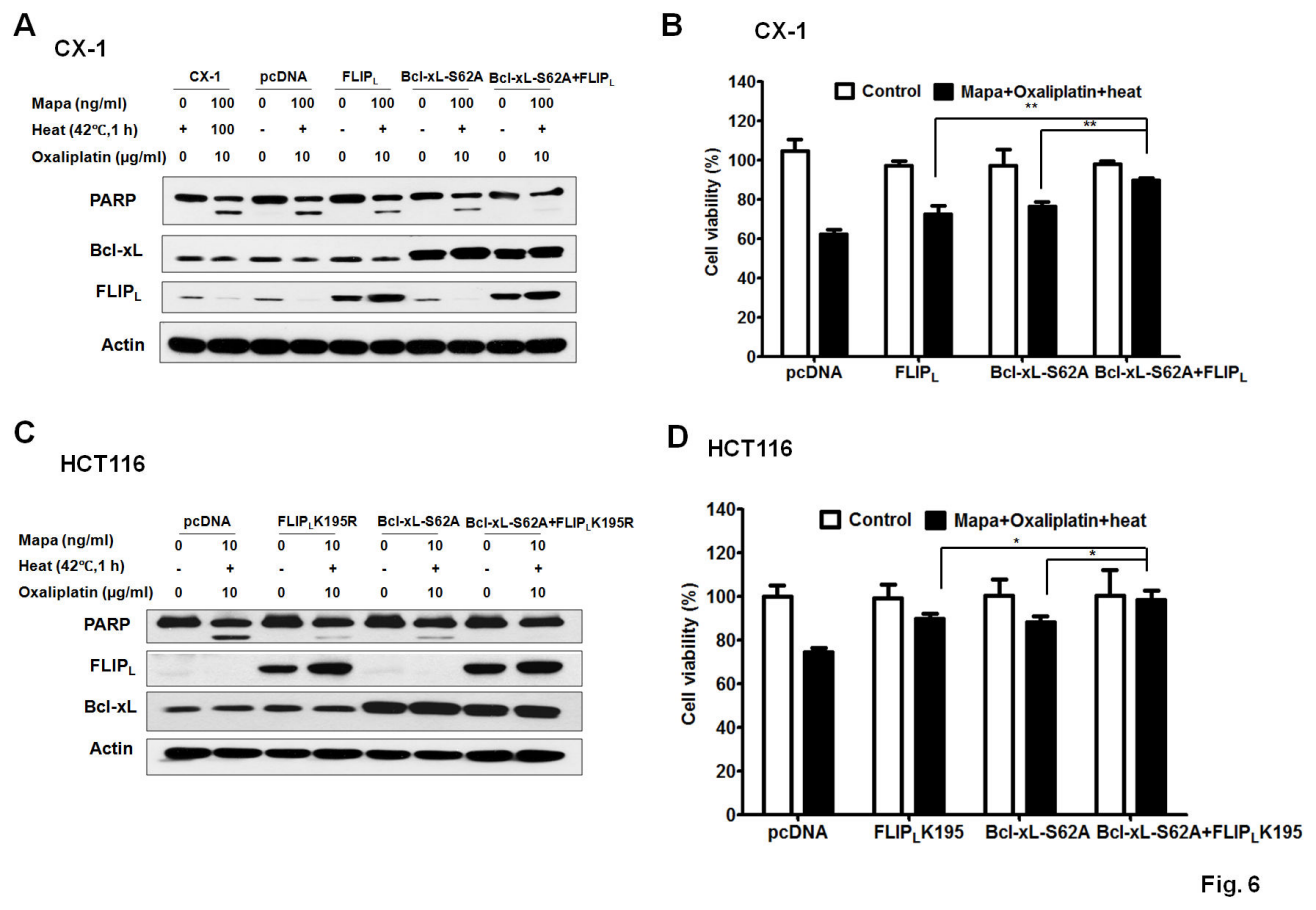
Protection from multimodality treatment-induced apoptosis by overexpression of c-FLIP_L_ WT/K195R and Bcl-xL-S62A in CX-1 or HCT116 cells. (A) CX-1 cells stably overexpressed with pcDNA, c-FLIP_L_ WT, Bcl-xL-S62A and c-FLIP_L_ WT + Bcl-xL-S62A, and three stable clones were pooled, and cells were heated at 42°C for 1 h in the presence/absence of Mapa (10 ng/ml) and oxaliplatin (10 µg/ml) and then incubated at 37^°^C for 3 h. The cleavage of PARP, and the level of c-FLIP_L_ and Bcl-xL were detected by Western blot analysis. (B) Cell viability was analyzed by MTS assay 24 h after treatment. Error bars represent SD from triplicate experiments. Asterisk ** represents a statistically significant difference (P <0.01). (C) HCT116 cells were transiently transfected with equal amount of plasmid containing mock, c-FLIP_L_ K195R, Bcl-xL-S62A and c-FLIP_L_ K195R + Bcl-xL-S62A. After 48 h incubation, cells were heated at 42°C for 1 h in the presence/absence of Mapa (10 ng/ml) and oxaliplatin (10 µg/ml) and then incubated at 37^°^C for 3 h. The cleavage of PARP, and the level of c-FLIP_L_ and Bcl-xL were detected by Western blot analysis. (D) Cell viability was analyzed by MTS assay 24 h after treatment. Error bars represent SD from triplicate experiments. Asterisk * represents a statistically significant difference (P <0.05).

Finally, we compared the effect of the multimodality treatment in CX-1 and HCT116 cells overexpressed with c-FLIP_L_ WT, Bcl-xL-S62A, Bcl-xL-S62A + c-FLIP_L_ WT (Figure 6A and 6B) and c-FLIP_L_ K195R, Bcl-xL-S62A, Bcl-xL-S62A +c-FLIP_L_ K195R (Figure 6C and 6D). We observed that c-FLIP_L_ WT/K195R or Bcl-xL-S62A partially blocked the effect of the multimodality treatment. Of note, the multimodality treatment-induced apoptosis was almost completely blocked by overexpression of both c-FLIP _L_WT/K195R and Bcl-xL-S62A (Figure 6A and 6C), indicating c-FLIP_L_ and Bcl-xL were independent factors contributing to the synergistic effect of the multimodality treatment. Similar results were obtained in cell viability assay (Figure 6B and 6D). Our results suggest that (a) reduction in c-FLIP_L_ level and (b) Bcl-xL phosphorylation at Ser62 are both responsible for the synergistic induction of apoptosis of the clinically relevant multimodality treatment.

## Discussion

Our laboratory has focused on identifying strategies and mechanisms for thermal sensitization in an attempt to improve the clinical efficacy of IHP [19,29–31]. We previously developed the multimodality treatment (oxaliplatin pretreatment + Mapa + hyperthermia) for colorectal cancer hepatic metastases. In this study, we investigated the efficacy and the underlying mechanisms of the more clinically relevant simultaneous treatment schedule of oxaliplatin + Mapa + hyperthermia-induced apoptosis and proposed that (a) reduction in c-FLIP_L_ level and (b) Bcl-xL phosphorylation at Ser62 are both responsible for the synergistic induction of apoptosis of the clinically relevant multimodality treatment.

First, we compared the efficacy of the pretreatment of oxaliplatin followed by Mapa and hyperthermia to that of simultaneous treatment of oxaliplatin, Mapa and hyperthermia (Figure S1). We observed that oxaliplatin pretreatment resulted in maximum apoptotic cell death which was consistent with our previous paper [19]. Notably, synergistic effect was still observed in the more clinically relevant multimodality treatment schedule (cotreatment with oxaliplatin). Assays of caspase inhibitors confirmed that both pathways played an important role in the synergistic effect of the multimodality treatment. It is known that combinatorial drug effects are complex, even for relatively specific drugs [32]. The goal of this study is to reveal the key molecules in mediating the synergistic induction of apoptosis and how these molecules rewired the apoptotic signaling networks.

Bcl-xL is a pro-survival member of the Bcl-2 family that plays indispensable roles in the intrinsic pathway. It is overexpressed in many malignant tumors including colorectal cancer. The status of Bcl-xL protein expression might be an independent prognostic marker for colorectal cancer patients [33]. Bcl-xL undergoes phosphorylation in response to microtubule inhibitors and other apoptotic stimuli [17,34]. Our study revealed that the oxaliplatin + Mapa + hyperthermia clinically relevant schedule synergistically induced Bcl-xL phosphorylation. We also observed that Bcl-xL phosphorylation required activated JNK, which can recognize a proline residue on the carboxyl side of the phospho-acceptor [35]. Our data highlighted the role of phosphorylation at Ser62, which antagonized the anti-apoptotic function, probably due to a changed interaction between Bax/phospho-mimic Bcl-xL-S62D and Bax/Bcl-xL-S62A. However, our data also implied Bcl-xL phosphorylation is crucial but not sufficient to the contribution of the synergistic effect of the multimodality treatment, indicating that other mechanisms should be identified.

c-FLIP is the major inhibitor of the extrinsic apoptotic pathway through inhibition of caspase-8 activation and processing at the death-inducing signaling complex (DISC) [36–40]. Differential splicing gives rise to long (c-FLIP_L_) and short (c-FLIP_S_) forms of c-FLIP. Both c-FLIP splice variants bind to FADD within the DISC. They compete with caspase 8 for DISC association and can form heteromeric complexes, thereby inhibiting apoptosis [37,41]. c-FLIP_L_, which is the most abundant isoform in many cancer cell lines, is a key regulator of colorectal cancer cell death and associated with a poor prognosis in colorectal cancer patients [39,42,43]. Given the central role of c-FLIP_L_ in extrinsic apoptotic death in colon cancer cells, we investigated in depth the mechanism of FLIP_L_ down-regulation in the multimodality treatment.

It is reported that c-FLIP is regulated at the transcriptional, translational level or through protein degradation [44–47]. We observed that the level of c-FLIP_L_ was reduced after hyperthermia. Quantitative RT-PCR showed that the decrease of c-FLIP level was not due to transcriptional regulation. FLIP_L_ was dramatically reduced in the presence of the protein synthesis inhibitor cycloheximide, indicating that c-FLIP_L_ was degraded during hyperthermia. Ubiquitination assay showed that endogenous c-FLIP_L_ underwent proteasomal-mediated degradation after hyperthermia. However, c-FLIP_L_ was a fast-turnover protein and resynthesized after incubation at 37°C. Interestingly, the level of c-FLIP_L_ remained decreased when hyperthermia was combined with oxaliplatin. Protein synthesis assay showed 46% protein synthesis inhibition in oxaliplatin combined with hyperthermia; thus the decrease of c-FLIP_L_ was due to the degradation through c-FLIP_L_ ubiquitination by hyperthermia and delay of c-FLIP_L_ restoration through protein synthesis inhibition by oxaliplatin combined with hyperthermia. We also found that c-FLIP_L_ K195R was refractory to degradation by hyperthermia and prevented the multimodality treatment-induced apoptotic cell death.

Our results showed that c-FLIP_L_ WT/K195R and Bcl-xL-S62A were independent factors that blocked the synergistic effect of the clinically relevant multimodality treatment, indicating that two modes of synergistic induction of apoptosis were involved in the multimodality treatment. The levels of Bcl-xL/c-FLIP_L_ may serve as biomarkers for multimodality treatment and prognosis. A high value of Bcl-xL/c-FLIP_L_ signature in the tumor may predict less response to the chemotherapy and thus the multimodality treatment would be suggested in this situation. Indeed several researchers reported that the prognosis was reversely correlated to the value of Bcl-xL/c-FLIP_L_ [33,43,48,49].

Taken together, we document here that the clinically relevant multimodality treatment oxaliplatin, Mapa and hyperthermia increased apoptosis signaling via both the intrinsic and extrinsic apoptotic pathways. Given the facts that hyperthermia has a favorable safety profile, oxaliplatin is a commonly used chemotherapeutic drug for colon cancers, and Mapa currently is undergoing clinical testing, this multimodality treatment has an excellent translational potential and should be considered for colorectal hepatic metastases treatment in clinics.

## Supporting Information

Figure S1 **Effect of pretreatment of oxaliplatin on multimodality-induced apoptosis.** CX-1 cells were pretreated with oxaliplatin for various times (0 h-20 h) and exposed to normothermic or hyperthermic (42°C) conditions for 1 h in the presence/absence of Mapa and oxaliplatin and then incubated for 3 h at 37°C. After treatment, the cleavage of PARP was detected by immunoblotting. Actin was used as loading control.
(TIF)

## References

[1] Ruers T, Bleichrodt RP (2002) Treatment of liver metastases, an update on the possibilities and results. Eur J Cancer 38: 1023-1033. doi:10.1016/S0959-8049(02)00059-X. PubMed: 11978527. 11978527 10.1016/s0959-8049(02)00059-x

[2] Jemal A, Bray F, Center MM, Ferlay J, Ward E et al. (2011) Global cancer statistics. CA Cancer J Clin 61: 69-90. doi:10.3322/caac.20107. PubMed: 21296855. 21296855 10.3322/caac.20107

[3] Zeh HJ 3rd, Brown CK, Holtzman MP, Egorin MJ, Holleran JL et al. (2009) A phase I study of hyperthermic isolated hepatic perfusion with oxaliplatin in the treatment of unresectable liver metastases from colorectal cancer. Ann Surg Oncol 16: 385-394. doi:10.1245/s10434-008-0179-5. PubMed: 19034580. 19034580 10.1245/s10434-008-0179-5

[4] Alexander HR Jr., Libutti SK, Pingpank JF, Bartlett DL, Helsabeck C et al. (2005) Isolated hepatic perfusion for the treatment of patients with colorectal cancer liver metastases after irinotecan-based therapy. Ann Surg Oncol 12: 138-144. doi:10.1245/ASO.2005.05.003. PubMed: 15827794. 15827794 10.1245/ASO.2005.05.003

[5] Varghese S, Xu H, Bartlett D, Hughes M, Pingpank JF et al. (2010) Isolated hepatic perfusion with high-dose melphalan results in immediate alterations in tumor gene expression in patients with metastatic ocular melanoma. Ann Surg Oncol 17: 1870-1877. doi:10.1245/s10434-010-0998-z. PubMed: 20221901. 20221901 10.1245/s10434-010-0998-z

[6] Hafström LR, Holmberg SB, Naredi PL, Lindnér PG, Bengtsson A et al. (1994) Isolated hyperthermic liver perfusion with chemotherapy for liver malignancy. Surg Oncol 3: 103-108. doi:10.1016/0960-7404(94)90005-1. PubMed: 7952389. 7952389 10.1016/0960-7404(94)90005-1

[7] Mom CH, Verweij J, Oldenhuis CN, Gietema JA, Fox NL et al. (2009) Mapatumumab, a fully human agonistic monoclonal antibody that targets TRAIL-R1, in combination with gemcitabine and cisplatin: a phase I study. Clin Cancer Res 15: 5584-5590. doi:10.1158/1078-0432.CCR-09-0996. PubMed: 19690193. 19690193 10.1158/1078-0432.CCR-09-0996

[8] Younes A, Vose JM, Zelenetz AD, Smith MR, Burris HA et al. (2010) A Phase 1b/2 trial of mapatumumab in patients with relapsed/refractory non-Hodgkin’s lymphoma. Br J Cancer 103: 1783-1787. doi:10.1038/sj.bjc.6605987. PubMed: 21081929. 21081929 10.1038/sj.bjc.6605987 PMC3008610

[9] Vulfovich M, Saba N (2005) Technology evaluation: mapatumumab, Human Genome Sciences/GlaxoSmithKline/Takeda. Curr Opin Mol Ther 7: 502-510. PubMed: 16248286. 16248286

[10] Trarbach T, Moehler M, Heinemann V, Köhne CH, Przyborek M et al. (2010) Phase II trial of mapatumumab, a fully human agonistic monoclonal antibody that targets and activates the tumour necrosis factor apoptosis-inducing ligand receptor-1 (TRAIL-R1), in patients with refractory colorectal cancer. Br J Cancer 102: 506-512. doi:10.1038/sj.bjc.6605507. PubMed: 20068564. 20068564 10.1038/sj.bjc.6605507 PMC2822942

[11] Greco FA, Bonomi P, Crawford J, Kelly K, Oh Y et al. (2008) Phase 2 study of mapatumumab, a fully human agonistic monoclonal antibody which targets and activates the TRAIL receptor-1, in patients with advanced non-small cell lung cancer. Lung Cancer 61: 82-90. doi:10.1016/j.lungcan.2007.12.011. PubMed: 18255187. 18255187 10.1016/j.lungcan.2007.12.011

[12] Zhang L, Fang B (2005) Mechanisms of resistance to TRAIL-induced apoptosis in cancer. Cancer Gene Ther 12: 228-237. doi:10.1038/sj.cgt.7700792. PubMed: 15550937. 15550937 10.1038/sj.cgt.7700792

[13] Dimberg LY, Anderson CK, Camidge R, Behbakht K, Thorburn A et al. (2013) On the TRAIL to successful cancer therapy? Predicting and counteracting resistance against TRAIL-based therapeutics. Oncogene 32: 1341-1350. doi:10.1038/onc.2012.164. PubMed: 22580613. 22580613 10.1038/onc.2012.164 PMC4502956

[14] Mahalingam D, Oldenhuis CN, Szegezdi E, Giles FJ, de Vries EG et al. (2011) Targeting TRAIL towards the clinic. Curr Drug Targets 12: 2079-2090. doi:10.2174/138945011798829357. PubMed: 21777191. 21777191 10.2174/138945011798829357

[15] Hegewisch-Becker S, Gruber Y, Corovic A, Pichlmeier U, Atanackovic D et al. (2002) Whole-body hyperthermia (41.8 degrees C) combined with bimonthly oxaliplatin, high-dose leucovorin and 5-fluorouracil 48-hour continuous infusion in pretreated metastatic colorectal cancer: a phase II study. Ann Oncol 13: 1197-1204. doi:10.1093/annonc/mdf216. PubMed: 12181242. 12181242 10.1093/annonc/mdf216

[16] Raymond E, Faivre S, Chaney S, Woynarowski J, Cvitkovic E (2002) Cellular and molecular pharmacology of oxaliplatin. Mol Cancer Ther 1: 227-235. PubMed: 12467217. 12467217

[17] El Fajoui Z, Toscano F, Jacquemin G, Abello J, Scoazec JY et al. (2011) Oxaliplatin sensitizes human colon cancer cells to TRAIL through JNK-dependent phosphorylation of Bcl-xL. Gastroenterology 141: 663-673. doi:10.1053/j.gastro.2011.04.055. PubMed: 21683075. 21683075 10.1053/j.gastro.2011.04.055

[18] Atallah D, Marsaud V, Radanyi C, Kornprobst M, Rouzier R et al. (2004) Thermal enhancement of oxaliplatin-induced inhibition of cell proliferation and cell cycle progression in human carcinoma cell lines. Int J Hyperthermia 20: 405-419. doi:10.1080/02656730310001637325. PubMed: 15204521. 15204521 10.1080/02656730310001637325

[19] Song X, Kim SY, Lee YJ (2012) The role of Bcl-xL in synergistic induction of apoptosis by mapatumumab and oxaliplatin in combination with hyperthermia on human colon cancer. Mol Cancer Res 10: 1567-1579. doi:10.1158/1541-7786.MCR-12-0209-T. PubMed: 23051936. 23051936 10.1158/1541-7786.MCR-12-0209-T PMC3528808

[20] Libutti SK, Barlett DL, Fraker DL, Alexander HR (2000) Technique and results of hyperthermic isolated hepatic perfusion with tumor necrosis factor and melphalan for the treatment of unresectable hepatic malignancies. J Am Coll Surg 191: 519-530. doi:10.1016/S1072-7515(00)00733-X. PubMed: 11085732. 11085732 10.1016/s1072-7515(00)00733-x

[21] Lee YJ, Galoforo SS, Battle P, Lee H, Corry PM et al. (2001) Replicating adenoviral vector-mediated transfer of a heat-inducible double suicide gene for gene therapy. Cancer Gene Ther 8: 397-404. doi:10.1038/sj.cgt.7700310. PubMed: 11498759. 11498759 10.1038/sj.cgt.7700310

[22] Cummins JM, Kohli M, Rago C, Kinzler KW, Vogelstein B et al. (2004) X-linked inhibitor of apoptosis protein (XIAP) is a nonredundant modulator of tumor necrosis factor-related apoptosis-inducing ligand (TRAIL)-mediated apoptosis in human cancer cells. Cancer Res 64: 3006-3008. doi:10.1158/0008-5472.CAN-04-0046. PubMed: 15126334. 15126334 10.1158/0008-5472.can-04-0046

[23] Odoux C, Fohrer H, Hoppo T, Guzik L, Stolz DB et al. (2008) A stochastic model for cancer stem cell origin in metastatic colon cancer. Cancer Res 68: 6932-6941. doi:10.1158/0008-5472.CAN-07-5779. PubMed: 18757407. 18757407 10.1158/0008-5472.CAN-07-5779 PMC2562348

[24] Gao W, Chen L, Ma Z, Du Z, Zhao Z et al. (2013) Isolation and Phenotypic Characterization of Colorectal Cancer Stem Cells with Organ-Specific Metastatic Potential. Gastroenterology. PubMed: 23747337. 10.1053/j.gastro.2013.05.049 23747337

[25] Liu C, Xue H, Lu Y, Chi B (2012) Stem cell gene Girdin: a potential early liver metastasis predictor of colorectal cancer. Mol Biol Rep 39: 8717-8722. doi:10.1007/s11033-012-1731-8. PubMed: 22714912. 22714912 10.1007/s11033-012-1731-8

[26] Zhao L, Yue P, Lonial S, Khuri FR, Sun SY (2011) The NEDD8-activating enzyme inhibitor, MLN4924, cooperates with TRAIL to augment apoptosis through facilitating c-FLIP degradation in head and neck cancer cells. Mol Cancer Ther 10: 2415-2425. doi:10.1158/1535-7163.MCT-11-0401. PubMed: 21914854. 21914854 10.1158/1535-7163.MCT-11-0401 PMC3237891

[27] Lin Y, Liu X, Yue P, Benbrook DM, Berlin KD et al. (2008) Involvement of c-FLIP and survivin down-regulation in flexible heteroarotinoid-induced apoptosis and enhancement of TRAIL-initiated apoptosis in lung cancer cells. Mol Cancer Ther 7: 3556-3565. doi:10.1158/1535-7163.MCT-08-0648. PubMed: 19001438. 19001438 10.1158/1535-7163.MCT-08-0648

[28] Song X, Kim SY, Zhou Z, Lagasse E, Kwon YT et al. (2013) Hyperthermia enhances mapatumumab-induced apoptotic death through ubiquitin-mediated degradation of cellular FLIP(long) in human colon cancer cells. Cell Death Dis 4: e577. doi:10.1038/cddis.2013.104. PubMed: 23559011. 23559011 10.1038/cddis.2013.104 PMC3641327

[29] Song X, Kim HC, Kim SY, Basse P, Park BH et al. (2012) Hyperthermia-enhanced TRAIL- and mapatumumab-induced apoptotic death is mediated through mitochondria in human colon cancer cells. J Cell Biochem 113: 1547-1558. PubMed: 22174016. 22174016 10.1002/jcb.24023 PMC3330147

[30] Alcala MA Jr., Park K, Yoo J, Lee DH, Park BH et al. (2010) Effect of hyperthermia in combination with TRAIL on the JNK-Bim signal transduction pathway and growth of xenograft tumors. J Cell Biochem 110: 1073-1081. doi:10.1002/jcb.22619. PubMed: 20544795. 20544795 10.1002/jcb.22619 PMC2967443

[31] Yoo J, Lee YJ (2008) Effect of hyperthermia and chemotherapeutic agents on TRAIL-induced cell death in human colon cancer cells. J Cell Biochem 103: 98-109. doi:10.1002/jcb.21389. PubMed: 17520700. 17520700 10.1002/jcb.21389

[32] Lee MJ, Ye AS, Gardino AK, Heijink AM, Sorger PK et al. (2012) Sequential application of anticancer drugs enhances cell death by rewiring apoptotic signaling networks. Cell 149: 780-794. doi:10.1016/j.cell.2012.03.031. PubMed: 22579283. 22579283 10.1016/j.cell.2012.03.031 PMC3501264

[33] Jin-Song Y, Zhao-Xia W, Cheng-Yu L, Xiao-Di L, Ming S et al. (2011) Prognostic significance of Bcl-xL gene expression in human colorectal cancer. Acta Histochem 113: 810-814. doi:10.1016/j.acthis.2011.01.002. PubMed: 21277008. 21277008 10.1016/j.acthis.2011.01.002

[34] Upreti M, Galitovskaya EN, Chu R, Tackett AJ, Terrano DT et al. (2008) Identification of the major phosphorylation site in Bcl-xL induced by microtubule inhibitors and analysis of its functional significance. J Biol Chem 283: 35517-35525. doi:10.1074/jbc.M805019200. PubMed: 18974096. 18974096 10.1074/jbc.M805019200 PMC2602892

[35] Ubersax JA, Ferrell JE Jr. (2007) Mechanisms of specificity in protein phosphorylation. Nat Rev Mol Cell Biol 8: 530-541. doi:10.1038/nrm2203. PubMed: 17585314. 17585314 10.1038/nrm2203

[36] Krueger A, Baumann S, Krammer PH, Kirchhoff S (2001) FLICE-inhibitory proteins: regulators of death receptor-mediated apoptosis. Mol Cell Biol 21: 8247-8254. doi:10.1128/MCB.21.24.8247-8254.2001. PubMed: 11713262. 11713262 10.1128/MCB.21.24.8247-8254.2001 PMC99990

[37] Lavrik IN, Krammer PH (2012) Regulation of CD95/Fas signaling at the DISC. Cell Death Differ 19: 36-41. doi:10.1038/cdd.2011.155. PubMed: 22075988. 22075988 10.1038/cdd.2011.155 PMC3252827

[38] Krueger A, Schmitz I, Baumann S, Krammer PH, Kirchhoff S (2001) Cellular FLICE-inhibitory protein splice variants inhibit different steps of caspase-8 activation at the CD95 death-inducing signaling complex. J Biol Chem 276: 20633-20640. doi:10.1074/jbc.M101780200. PubMed: 11279218. 11279218 10.1074/jbc.M101780200

[39] Longley DB, Wilson TR, McEwan M, Allen WL, McDermott U et al. (2006) c-FLIP inhibits chemotherapy-induced colorectal cancer cell death. Oncogene 25: 838-848. doi:10.1038/sj.onc.1209122. PubMed: 16247474. 16247474 10.1038/sj.onc.1209122

[40] Yu JW, Shi Y (2008) FLIP and the death effector domain family. Oncogene 27: 6216-6227. doi:10.1038/onc.2008.299. PubMed: 18931689. 18931689 10.1038/onc.2008.299

[41] Irmler M, Thome M, Hahne M, Schneider P, Hofmann K et al. (1997) Inhibition of death receptor signals by cellular FLIP. Nature 388: 190-195. doi:10.1038/40657. PubMed: 9217161. 9217161 10.1038/40657

[42] Wilson TR, McLaughlin KM, McEwan M, Sakai H, Rogers KM et al. (2007) c-FLIP: a key regulator of colorectal cancer cell death. Cancer Res 67: 5754-5762. doi:10.1158/0008-5472.CAN-06-3585. PubMed: 17575142. 17575142 10.1158/0008-5472.CAN-06-3585

[43] Ullenhag GJ, Mukherjee A, Watson NF, Al-Attar AH, Scholefield JH et al. (2007) Overexpression of FLIPL is an independent marker of poor prognosis in colorectal cancer patients. Clin Cancer Res 13: 5070-5075. doi:10.1158/1078-0432.CCR-06-2547. PubMed: 17785559. 17785559 10.1158/1078-0432.CCR-06-2547

[44] Mawji IA, Simpson CD, Gronda M, Williams MA, Hurren R et al. (2007) A chemical screen identifies anisomycin as an anoikis sensitizer that functions by decreasing FLIP protein synthesis. Cancer Res 67: 8307-8315. doi:10.1158/0008-5472.CAN-07-1687. PubMed: 17804746. 17804746 10.1158/0008-5472.CAN-07-1687

[45] Seo BR, Min KJ, Kim S, Park JW, Park WK et al. (2013) Anisomycin treatment enhances TRAIL-mediated apoptosis in renal carcinoma cells through the down-regulation of Bcl-2, c-FLIP(L) and Mcl-1. Biochimie 95: 858-865. doi:10.1016/j.biochi.2012.12.002. PubMed: 23261849. 23261849 10.1016/j.biochi.2012.12.002

[46] Bartke T, Siegmund D, Peters N, Reichwein M, Henkler F et al. (2001) p53 upregulates cFLIP, inhibits transcription of NF-kappaB-regulated genes and induces caspase-8-independent cell death in DLD-1 cells. Oncogene 20: 571-580. doi:10.1038/sj.onc.1204124. PubMed: 11313989. 11313989 10.1038/sj.onc.1204124

[47] Safa AR, Day TW, Wu CH (2008) Cellular FLICE-like inhibitory protein (C-FLIP): a novel target for cancer therapy. Curr Cancer Drug Targets 8: 37-46. doi:10.2174/156800908783497087. PubMed: 18288942. 18288942 10.2174/156800908783497087 PMC4524510

[48] Zhang YL, Pang LQ, Wu Y, Wang XY, Wang CQ et al. (2008) Significance of Bcl-xL in human colon carcinoma. World J Gastroenterol 14: 3069-3073. doi:10.3748/wjg.14.3069. PubMed: 18494061. 18494061 10.3748/wjg.14.3069 PMC2712177

[49] Korkolopoulou P, Saetta AA, Levidou G, Gigelou F, Lazaris A et al. (2007) c-FLIP expression in colorectal carcinomas: association with Fas/FasL expression and prognostic implications. Histopathology 51: 150-156. doi:10.1111/j.1365-2559.2007.02723.x. PubMed: 17559541. 17559541 10.1111/j.1365-2559.2007.02723.x

