# Supplementary figures and images for: Evidence for Two Modes of Synergistic Induction of Apoptosis by Mapatumumab and Oxaliplatin in Combination with Hyperthermia in Human Colon Cancer Cells
Source: PLoS One. 2013 Aug 27;8(8):e73654. doi: 10.1371/journal.pone.0073654 (PMC3754951; doi:10.1371/journal.pone.0073654)

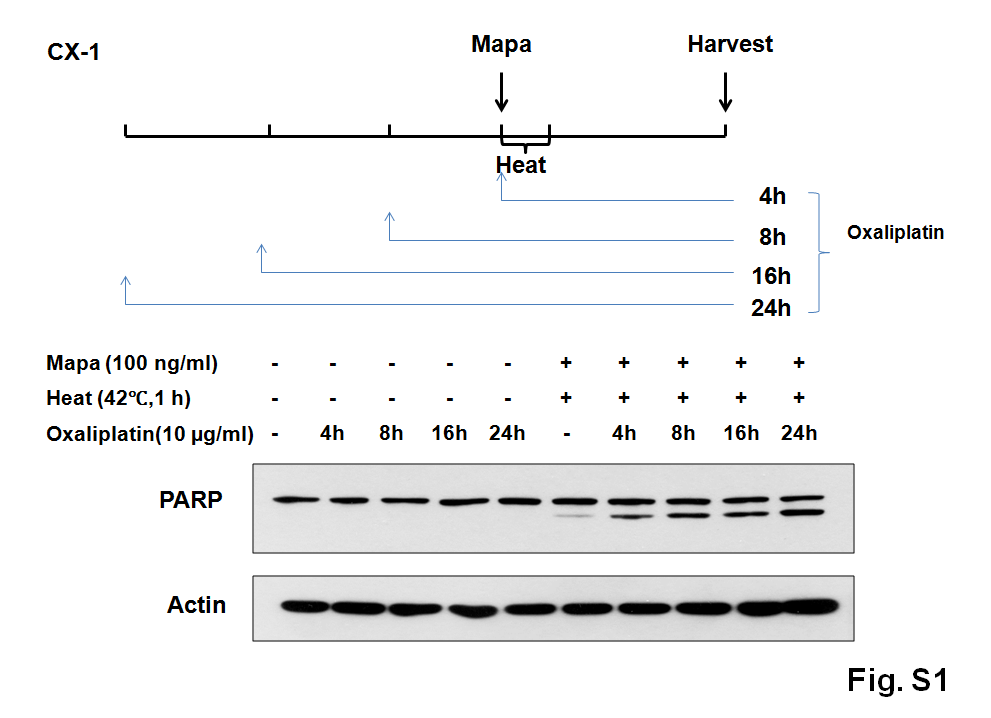

Supplement: Figure S1 — Effect of pretreatment of oxaliplatin on multimodality-induced apoptosis. CX-1 cells were pretreated with oxaliplatin for various times (0 h-20 h) and exposed to normothermic or hyperthermic (42°C) conditions for 1 h in the presence/absence of Mapa and oxaliplatin and then incubated for 3 h at 37°C. After treatment, the cleavage of PARP was detected by immunoblotting. Actin was used as loading control. (TIF) [file pone.0073654.s001.tif]
